# Dataset on the compounds from the leaves of Vietnamese *Machilus thunbergii* and their anti-inflammatory activity

**DOI:** 10.1016/j.dib.2023.109713

**Published:** 2023-10-22

**Authors:** Dao Cuong To, Le Minh Hoang, Hoa Thi Nguyen, Truong Thi Viet Hoa, Nhung Truong Thi Thuy, Manh Hung Tran, Phi Hung Nguyen, Phuong Dai Nguyen Nguyen, Ngu Truong Nhan, Nguyen Thi Thu Tram

**Affiliations:** a Phenikaa University Nano Institute (PHENA), Phenikaa University, Yen Nghia, Ha Dong, Hanoi 12116, Vietnam; b School of Medicine & Pharmacy, The University of Danang, Hoa Quy, Ngu Hanh Son, Da Nang City 550000, Vietnam; c Institute of Natural Products Chemistry, Vietnam Academy of Science and Technology (VAST), 18 Hoang Quoc Viet, Cau Giay, Hanoi 122100, Vietnam; d Tay Nguyen University, 567 Le Duan, Buon Ma Thuot, Dak Lak 630000, Vietnam; e Faculty of Basic Sciences, Can Tho University of Medicine and Pharmacy, 179 Nguyen Van Cu, An Khanh Ward, Ninh Kieu District, Can Tho 94000, Vietnam

**Keywords:** Flavonoid, Inflammation, NO production, Cytotoxic, RAW264.7 cells

## Abstract

*Machilus thunbergii* has a history of traditional applications including treating dyspepsia, apoplexy, headaches, abdominal pain, abdominal distension, and leg edema [1]. It is also employed for alleviating allergies, inflammation, pain relief, promoting blood circulation, addressing costal chondritis, and sinusitis [2]. Research into the chemical composition of *M. thunbergii* has revealed the presence of lignans, flavonoids, lactones, and essential oils [1,[3], [4], [5]. While some investigations have explored the inhibitory effects of extracts and lignan compounds from this species on NO production [6], [7], [8], there has been no research into the flavonoids isolated from this plant and their potential for inhibiting NO production, given our reachable referencing. The ethyl acetate (EtOAc) soluble fraction of *M. thunbergii* leaves was subjected to column chromatography (CC) using silica gel and Sephadex LH-20 for compound isolation. Nuclear magnetic resonance (NMR) data primarily facilitated the determination of isolated compound structures. Anti-inflammatory activity was evaluated against lipopolysaccharide (LPS)-induced nitric oxide (NO) production in macrophage RAW264.7 cells. Anti-inflammatory activity-guided fractionation led to the isolation of twelve secondary metabolites (**1**−**12**). The compounds were identified as quercetin (**1**), kaempferol (**2**), rhamnetin (**3**), quercitrin (**4**), hyperoside (**5**), reynoutrin (**6**), guaijaverin (**7**), afzelin (**8**), astragalin (**9**), rutin (**10**), kaempferol-3-O-rutinoside (**11**), and rhamnetin-3-O-rutinoside (**12)**. Compounds **3, 5, 6, 9, 11**, and **12** were isolated from *M. thunbergii* for the first time. Evaluation against LPS-induced NO production in macrophage RAW264.7 cells showed that **1−3** exhibited potent inhibitory activity with IC_50_ values of 15.45, 25.44, and 19.82 µM, respectively. Compounds **4−9** demonstrated IC_50_ values ranging from 42.15 to 67.42 µM, while **10−12** exhibited inactivity (IC_50_ > 100 µM).

Specifications Table

**Table:** 

Subject	Chemistry, Biochemistry
Specific subject area	Isolating and elucidating the structures of the isolated compounds, along with the assessment of their ability to inhibit NO production
Data format	Raw, Analyzed
Type of data	Table, Figure
Data collection	The leaves of *M. thunbergii* were gathered from Hoa Binh province, Vietnam, in February 2021. Compounds were isolated by using column chromatography (CC), the following materials were utilized: silica gel (Si 60 F254, 40-63 mesh, Merck, St. Louis, MO, USA), YMCGEL (ODS-A, 12 nm S-150 µm, YMC Co., Ltd., Kyoto, Japan), and Sephadex LH-20 (Sigma–Aldrich, MO, USA). The chemical structure of isolated compounds was determined by nuclear magnetic resonance (NMR) data primarily. The cytotoxic assay was conducted by MTS assay [9]. The inhibition of NO production assay was determined using the Griess reaction [10].
Data source location	• Institution: Phenikaa University Nano Institute (PHENA), Phenikaa University • City/Town/Region: Hanoi, Ha Dong, Yen Nghia • Country: Vietnam
Data accessibility	Repository name: Mendeley Data Data identification number: 10.17632/j26n3rjbv6.1 Direct URL to data: https://data.mendeley.com/datasets/j26n3rjbv6/1 Instructions for accessing these data: NMR data of isolated compounds

## 1 Value of the Data

• *M. thunbergii* is a medicinal plant, which contains extracts and lignan compounds on NO production inhibition. However, the NO production inhibition of flavonoids has not yet been evaluated.
• This study aimed to explore the chemical constituents of Vietnamese *M. thunbergii* and assess their inhibitory activity against NO production.
• This study provides valuable information on the flavonoid constituents from the leaves of *M. thunbergii*. The compounds were identified as quercetin (**1**), kaempferol (**2**), rhamnetin (**3**), quercitrin (**4**), hyperoside (**5**), reynoutrin (**6**), guaijaverin (**7**), afzelin (**8**), astragalin (**9**), rutin (**10**), kaempferol-3-O-rutinoside (**11**), and rhamnetin-3-O-rutinoside (**12)**. Compounds **3, 5, 6, 9, 11**, and **12** were isolated from *M. thunbergii* for the first time. The plant's chemical profile presented here could facilitate the advancement of high-quality herbal medicinal products.
• This study also presented detailed data for cytotoxic and anti-inflammatory activities.
• The findings imply that *M. thunbergii* and its natural secondary metabolites could offer anti-inflammatory effects through NO inhibition.

## 2 Data Description

**Fig. 1:**
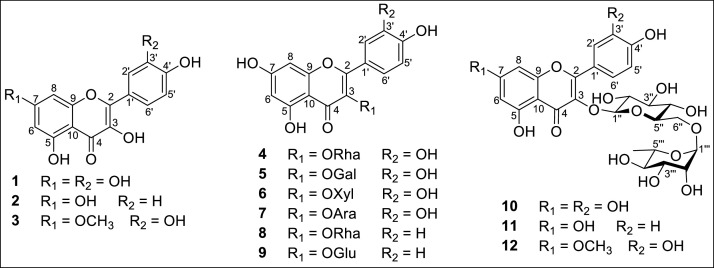
Structure of compounds **1**−**12** from *M. thunbergii.*

**Fig. 2:**
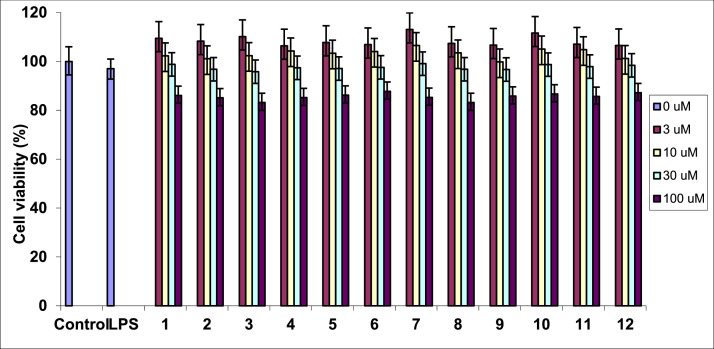
Effect on cell viability by LPS stimulation in the presence of compounds **1**−**12**. Cell viability was determined by MTS assay and expressed as a percentage of the control without the addition of indicated compounds **1**−**12**.

**Fig. 3:**
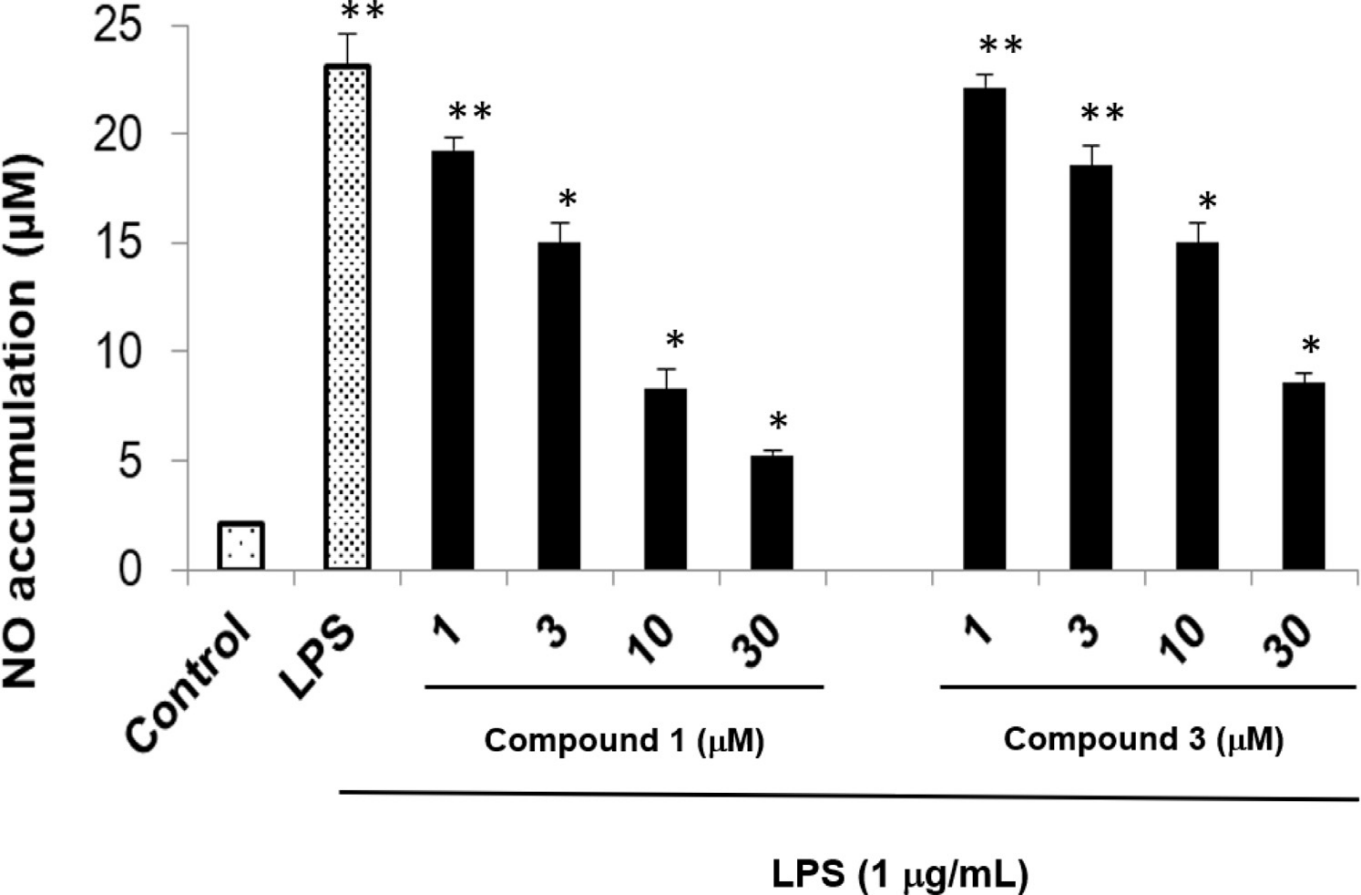
Inhibitory effect of compounds **1** and **3** on the LPS-induced NO production in RAW264.7 cells. RAW264.7 cells were pre-treated with various concentrations (1, 3, 10, and 30 µM) of the tested compounds for 1 hour, followed by treatment with LPS (1 µg/mL), and then incubated for 24 hours. Control values were obtained in the absence of both LPS and the compound. The blank group was used as 0.1% DMSO-treated cells. Data are presented as the mean ± SD of results from three independent experiments (*p <0.01; **p < 0.05).

The dataset in this article contains full extraction and isolation and NMR data of compounds isolated from *M. thunbergii* as tabulated in Fig. 1 and Supplementary data file. Furthermore, this article also identified the chemical stucture of isolated compounds from *M. thunbergii*, Fig. 1, and Supplementary data file. Effect on cell viability by LPS stimulation in the presence of compounds was determined by MTS assay and expressed as a percentage of the control without the addition of indicated compounds, Fig. 2. To assess the inhibitory activity on NO production, RAW 264.7 cells were exposed to varying concentrations (1–30 µM) of the isolated compounds, and the level of NO production was determined using the Griess reaction as illustrated in Table 1. Inhibitory effect of compounds **1** and **3** on the LPS-induced NO production in RAW264.7 cells. RAW264.7 cells were pre-treated with various concentrations (1, 3, 10, and 30 µM) of the tested compounds for 1 hour, followed by treatment with LPS (1 µg/mL), and then incubated for 24 hours. Control values were obtained in the absence of both LPS and the compounds, Fig. 3.

**Table 1:** NO production inhibition in RAW264.7 cells of isolated compounds (**1**−**12**).

Compounds	IC_50_ value (µM)^a^
1	15.45 ± 1.72
2	25.44 ± 1.81
3	19.82 ± 1.54
4	43.87 ± 3.98
5	45.68 ± 3.65
6	46.56 ± 2.77
7	42.15 ± 2.82
8	64.28 ± 2.05
9	67.42 ± 3.79
10	> 100
11	> 100
12	> 100
Sappanone A^b^	8.32 ± 0.04

a The inhibitory effects are represented as the molar concentration (µM) giving 50% inhibition (IC_50_) relative to the vehicle control. Values are mean ± S.D (*n* = 3);

b Positive control.

### 2.1 Chemical structure identifications

The MeOH extract was partitioned with *n*-hexane and EtOAc to yield *n*-hexane and EtOAc-soluble fractions. This fraction was subsequently subjected to CC using silica gel and Sephadex LH-20 to isolate twelve secondary metabolites (**1**–**12**) (Fig. 1 and Supplementary data).

The ^1^H-NMR spectrum of compounds **1**–**3** displayed characteristic signals due to aromatic protons of two benzene rings, along with those of hydroxyl groups at C-3, C-5, and C-4′. Their ^13^C-NMR spectrum revealed the signals of oxygen-substituted carbons at C-2 and C-9, as well as two quaternary carbons at C-10 and C-1′ (Fig. 1 and Supplementary Materials). The presence of an olefinic group [*δ*_C_ 150.1-158.0 (C-2) and *δ*_C_ 137.5-138.4 (C-3)] and a ketone carbon at C-4 [*δ*_C_ 177.5-177.9 (C-4)] in the ^1^H- and ^13^C-NMR spectra indicated **1**−**3** to be flavonol [11]. Detailed analysis of the ^1^H- and ^13^C-NMR spectra of **1** revealed that **1** possessed five aromatic protons (H-6, H-8, H-2′, H-5′, and H-6′) and seven oxygenated carbons (C-2, C-3, C-5, C-7, C-9, C-3′, and C-4′). Compound **2** possessed six oxygenated carbons (C-2, C-3, C-5, C-7, C-9, and C-4′) and showed six aromatic protons (H-6, H-8, H-2′, H-3′, H-5′, and H-6′). Compound **3** also possessed five aromatic protons (H-6, H-8, H-2′, H-5′, and H-6′) but showed a methoxy carbon at C-7 (Fig. 1 and Supplementary Materials). Therefore, by comparing the ^1^H- and ^13^C-NMR data of these compounds with those published in the literature, compounds **1**–**3** were identified as quercetin (**1**) [11], kaempferol (**2**) [11], and rhamnetin (**3**) [12], respectively. This is the first reported identification of rhamnetin (**3**) from *M. thunbergii.*

Compounds **4**–**9** were isolated as yellow powder. The presence of an olefinic group [*δ*_C_ 158.1–159.3 (C-2) and *δ*_C_ 135.6–136.5 (C-3)], along with a ketone carbon at C-4 (*δ*_C_ 179.4–179.8) in the ^1^H- and ^13^C-NMR spectra indicated that compounds **4**–**9** were flavonols [11]. The ^1^H- and ^13^C-NMR spectra of compounds **4**–**7** were similar to those of compound **1**, except for the replacement of the hydroxyl group at C-3 in **1** with a rhamnose unit in **4**, a galactose unit in **5**, a xylose unit in compound **6**, and an arabinose unit in compound **7** (Fig. 1 and Supplementary Materials). On the other hand, the ^1^H- and ^13^C-NMR spectra of compounds **8** and **9** were akin to those of compound **2**, but with the substitution of the hydroxyl group at C-3 in compound **2** by a rhamnose unit in compound **8** and a glucose unit in compound **9** (Fig. 1 and Supplementary Materials). By comparing the ^1^H- and ^13^C-NMR data of these compounds with those published in the literature, compounds **4**–**9** were identified as quercitrin (**4**) [13], hyperoside (**5**) [14], reynoutrin (**6**) [13], guaijaverin (**7**) [13], afzelin (**8**) [12], and astragalin (**9**) [15], respectively. This study marks the first identification of hyperoside (**5**) and reynoutrin (**6**) from *M. thunbergii* as well.

Compounds **10**–**12** were isolated as yellow amorphous powder. The presence of an olefinic group [*δ*_C_ 158.6–159.6 (C-2) and *δ*_C_ 158.6 135.6–135.8 (C-3)], along with a ketone carbon at C-4 (*δ*_C_ 158.6 179.5–179.6) in the ^1^H- and ^13^C-NMR spectra indicated that compounds **10**–**12** were flavonols [11]. The ^1^H- and ^13^C-NMR spectra of compound **10** were similar to those of compound **1**, except for the substitution of the hydroxyl group at C-3 in **1** with a rutinose unit (*α*-L-rhamnopyranosyl-(1→6)-*β*-D-glucopyranose) in **10**. Likewise, the ^1^H- and ^13^C-NMR spectra of compound **11** were akin to those of compound **2**, but with the replacement of the hydroxyl group at C-3 in compound **2** by a rutinose unit in compound **11** (Fig. 1 and Supplementary Materials). On the other hand, the ^1^H- and ^13^C-NMR spectra of compound **12** were also reminiscent of those of compound **3**, except for the substitution of the hydroxyl group at C-3 in compound 3 by a rutinose unit in compound **12** (Fig. 1 and Supplementary data). Consequently, compounds **10**–**12** were identified as rutin (**10**) [13], kaempferol-3-O-rutinoside (**11**) [13], and rhamnetin-3-O-rutinoside (**12**) [16], respectively. This study marks the first identification of kaempferol-3-O-rutinoside (**11**) and rhamnetin-3-O-rutinoside (**12**) from *M. thunbergii* as well.

### 2.2 Anti-inflammatory activity

In the initial experiment, a cytotoxic assay was conducted to establish the safe and non-toxic concentrations of the isolated compounds (**1**–**12**) for the subsequent assays. The non-toxic nature of the isolated compounds was demonstrated by the maintenance of over 90% cell viability as determined by the MTS assay [9]. Notably, the isolated compounds exhibited toxicity to RAW 264.7 cells at a concentration of 100 µM. Consequently, this concentration was excluded from the treatment range, and concentrations ranging from 1 to 30 µM were selected for further investigation, Fig. 2.

To assess the inhibitory activity on NO production, RAW 264.7 cells were exposed to varying concentrations (1–30 µM) of the isolated compounds, and the level of NO production was determined using the Griess reaction [10]. As illustrated in Table 1, compounds **1**–**3** displayed the most potent inhibitory activity against LPS-induced NO production, showcasing IC_50_ values of 15.45, 25.44, and 19.82 µM, respectively. Following this, compounds **4**–**9** exhibited IC_50_ values ranging from 43.87 to 67.42 µM, respectively, while compounds **10**–**12** demonstrated inactivity (IC_50_ > 100 µM). As a reference, sappanone A was employed as a positive inhibitor and notably suppressed LPS-induced NO production with an IC_50_ value of 8.32 µM.

The control group did not receive either LPS or the samples. Consequently, the observed inhibitory effects of these compounds on NO production were not influenced by any cytotoxic impact. Upon stimulation with LPS (1 µg/mL), the control group exhibited an approximately 11-fold increase in NO production after 24 h. In contrast, compounds **1** and **3** demonstrated a dose-dependent reduction in NO production 24 h following LPS stimulation, Fig. 3.

Macrophages, known for their role in inflammatory pathways, release various mediators such as proinflammatory cytokines, hydrolytic enzymes, growth factors, cytotoxic cytokines, and nitric oxide (NO) [17]. The transcription of activated macrophages induces the expression of inducible nitric oxide synthase (iNOS), responsible for generating NO from L-arginine. An excessive output of NO by iNOS can lead to detrimental effects like septic shock and inflammatory disorders. Therefore, assessing NO production induced by lipopolysaccharide (LPS) through iNOS inhibition provides insight into the inflammatory process's modulation [18]. In our study, compound **1**, the flavanonol quercetin, exhibited the strongest inhibition of NO production (IC_50_ = 15.45 µM) among the isolated compounds. This effect could be attributed to the presence of 3,4-hydroxylation in the benzene ring, similar to previous reports [19,20]. Compounds **2** and 3 showed reduced inhibitory activities (IC_50_ values of 25.44 and 19.82 µM, respectively), likely due to the absence of 3-hydroxylation (compound **2**) or the presence of methoxy groups (compound **3**) in the benzene ring [10,20]. Compounds **4**−**7** displayed further reduced inhibitory activities (IC_50_ values ranging from 42.15 to 46.56 µM) compared to compounds **1**−**3**, possibly due to the sugar units (rhamnose, galactose, xylose, and arabinose) despite the presence of 3,4-hydroxylation in the benzene ring. Compounds **8** and **9** exhibited even greater reduction in inhibitory activities (IC_50_ values of 64.28 and 67.42 µM) due to the combination of sugar units (rhamnose and glucose) and the lack of 3-hydroxylation in the benzene ring. Lastly, compounds **10**−**12** were inactive (IC_50_ > 100 µM) due to the presence of multiple sugar units (rutinose unit) combined with the absence of 3-hydroxylation (compound **11**) or the presence of methoxy groups (compound **12**) in the benzene ring [10,20].

## 3 Experimental Design, Materials and Methods

### 3.1 General experimental procedure

The ^1^H NMR (400 MHz) and ^13^C NMR (100 MHz) spectra were acquired using a Varian Unity Inova 400 MHz spectrometer (Varian, Inc., California, USA), while the 1H NMR (500 MHz) and 13C NMR (125 MHz) spectra were obtained using a Bruker Avance 500 MHz spectrometer (Bruker Daltonics, Ettlingen, Germany). For column chromatography (CC), the following materials were utilized: silica gel (Si 60 F254, 40-63 mesh, Merck, St. Louis, MO, USA), YMCGEL (ODS-A, 12 nm S-150 µm, YMC Co., Ltd., Kyoto, Japan), and Sephadex LH-20 (Sigma–Aldrich, MO, USA). All solvents underwent redistillation before use. Analytical purposes involved the use of pre-coated silica gel 60 F254 and RP-C18 F254S (Merck, Darmstadt, Germany) TLC plates. For visualization, compounds were exposed to UV radiation (254 nm and 365 nm) and also subjected to spraying with 10% H2SO4, followed by heat application using a heat gun.

### 3.2 Plant material

The leaves of *M. thunbergii* were gathered from Hoa Binh province, Vietnam, in February 2021. Botanical identification was expertly conducted by Nguyen Quoc Binh, Ph.D., of the Vietnam National Museum of Nature, Vietnam Academy of Science and Technology (VAST). To ensure traceability, a voucher specimen (MT-L-1610) was meticulously archived at the Natural Product Research and Development Lab, Phenikaa University, Vietnam.

### 3.3 Extraction and isolation

The leaves of *M. thunbergii* (5.0 kg) were subjected to reflux extraction using methanol (MeOH) for 1 hour. The resulting extract was then filtered and subsequently evaporated under reduced pressure, yielding a crude MeOH extract. This MeOH extract (195 g) was then dissolved in hot water and partitioned successively with *n*-hexane and EtOAc. The resultant fractions were *n*-hexane (45 g), EtOAc (95 g), and water (H2O). An activity-guided fractionation process led to the selection of the EtOAc fraction for further investigation. The EtOAc soluble fraction (95 g) was subjected to chromatography on a silica gel column chromatography (CC) using a stepwise gradient of CH_2_Cl_2_-MeOH (100:1 to 0:1, each 2.0 L), resulting in the isolation of twenty fractions (E1 – E20) based on their TLC profiles. Sub-fraction E9 (1.5 g) was then subjected to a further round of chromatography on a silica gel column, eluting with a gradient of CH_2_Cl_2_-EtOAc (10:1 to 3:1), yielding five fractions (E9.1 - 9.5). Sub-fraction E9.3 (500 mg) was further purified using ODS silica gel CC, employing a gradient of MeOH:H_2_O (1:2 to 2:1), leading to the isolation of compounds **2** (60 mg) and **3** (25 mg). Sub-fraction E9.4 (200 mg) underwent purification using ODS silica gel CC, with acetonitrile:H_2_O (1:2 to 1:1) as the eluent, resulting in the isolation of compound **1** (65 mg). Fraction 10 (6.5 g) was subjected to silica gel CC, utilizing CH_2_Cl_2_-acetone (10:1 to 1:1) as the eluent, yielding fifteen sub-fractions (E10.1 to 10.15). Sub-fraction E10.13 (400 mg) was further purified using ODS silica gel CC, employing a gradient of MeOH:H_2_O (1:2 to 2:1), and leading to the isolation of compounds **4** (25 mg) and **5** (36 mg). Sub-fraction E10.14 (150 mg) was purified using RP-C18 silica gel CC, with a gradient of acetonitrile:H_2_O (1:3 to 1:1) as the eluent, resulting in the isolation of compound **6** (15 mg). Fraction 11 (1.5 g) was subjected to ODS silica gel CC, using MeOH:H_2_O (1:3 to 2:1) as the eluent, leading to the isolation of eight sub-fractions (E11.1 to E11.8). Sub-fraction E11.3 (210 mg) underwent purification using Sephadex LH-20 CC, with a gradient of MeOH:H_2_O (1:1), yielding compounds **7** (22 mg) and **8** (25 mg). Further purification of sub-fraction E11.5 (100 mg) using Sephadex LH-20 CC, employing a gradient of MeOH:H_2_O (1:1), led to the isolation of compound **9** (25 mg). Fraction 14 (4.5 g) was subjected to RP-C18 silica gel CC, using MeOH:H_2_O (1:3 to 1:1) as the eluent, yielding ten sub-fractions (E14.1 to E14.10). Sub-fraction E14.4 (510 mg) was further purified using Sephadex LH-20 CC, with a gradient of MeOH:H_2_O (1:1), leading to the isolation of compounds **10** (52 mg) and **11** (35 mg). Further purification of sub-fraction E14.7 (150 mg) using Sephadex LH-20 CC, with a gradient of MeOH:H_2_O (1:1), resulted in the isolation of compound **12** (20 mg).

### 3.4 Cell culture

RAW264.7 cells were purchased from the American Type Culture Collection (Manssas, VA, USA) and were maintained in Dulbecco's Modified Essential. These cells were grown at 37 °C in DMEM supplemented with penicillin (100 units/mL), streptomycin (100 µg/mL), and 10% heat-inactivated fetal bovine serum (FBS, Cambrex, Charles City, IA, USA). The cells were maintained in a humidified 5% CO_2_ and atmosphere at 37°C. Cells were counted with a hemocytometer and the number of viable cells was determined by trypan blue dye exclusion.

### 3.5 Cell viability assay

The viability assay was evaluated by MTS [3-(4,5-dimethylthiazol-2-yl)-5-(3-carboxymethoxyphenyl)-2-(4-sulfophenyl)-2*H*-tetrazolium] assay [9]. Briefly, 100 µL cells (5 × 10^3^ cells per well) in medium (DMEM supplemented with 10% FBS and 1% penicillin-streptomycin) were plated in 96-well plate and incubated for 24 hours at 37 °C in a humidified atmosphere incubator with 5% CO_2_. The medium then washed and added with 99 µL new medium and 1 µL of each compound in different concentrations and DMSO in triplicate then the plate was incubated for 24 h. Untreated cells were served as the control. Briefly, 20 µL MTS was added to each well. The plate was incubated in 5% CO_2_ at 37 °C incubator for 4 h. The absorbance was measured at 490 nm on a microplate reader (Molecular Devices, Emax, Sunnyvale, CA, USA).

### 3.6 Determination of NO production

The level of NO production was determined by measuring the amount of nitrite from the cell culture supernatants as described previously [10]. The RAW 264.7 cells were seeded at a density of 1 × 10^5^ cells/well in 24 well plates and incubated for 12 h at 37 °C and 5% CO_2_. Then media of each well were aspirated and fresh FBS (Fetal Bovine Serum)-free DMEM (Dulbecco's Modified Eagle Medium) media were replaced. Different concentrations of isolated compounds (1, 3, 10 and 30 µM) were prepared in FBS-free DMEM to give a total volume of 500 µL in each well of a microtiter plate. After 1 h treatment, cells were stimulated with or without 1 µg/ml of LPS for 24 h. The quantity of nitrite in the culture medium was measured as an indicator of NO production. Amounts of nitrite, a stable metabolite of NO, were measured using Griess reagent (1% sulfanilamide and 0.1% naphthylethylenediamine dihydrochloride in 2.5% phosphoric acid). Briefly, 100 µL of cell culture medium was mixed with 100 µL of Griess reagent. Subsequently, the mixture was incubated at room temperature for 10 min and the absorbance at 540 nm was measured in a microplate reader (Biotek, Winooski, VT, USA). Fresh culture medium was used as a blank in every experiment. The quantity of nitrite was determined from a sodium nitrite (NaNO_2_) standard curve. For this experiment, Sappanone A was used as a positive control.

### 3.7 Statistical analysis

Data are presented as the mean ± standard deviation (SD). Graphs were generated, and statistical analyses were conducted using SigmaPlot 6.0 and SigmaStat 3.1 (Systat Software, San Jose, CA, USA). For the analysis, we employed ANOVA followed by the Tukey's test on pre-validated data. Alternatively, Prism (GraphPad Software, San Diego, CA, USA) was used for comparisons between two groups, utilizing an unpaired Student's t-test. For comparisons involving more than two groups, we used one-way nonparametric ANOVA with Tukey's test followed by Bonferroni post hoc analysis or correlation analysis, as appropriate. Statistical significance levels were set at ** *p* < 0.05; * *p* < 0.1.

## 4 Limitations

Although our study focused solely on the inhibitory activity of NO production, it did not evaluate other inflammatory agents such as prostaglandin E2 (PGE2) or proinflammatory cytokines like ILs, TNF-α, and IFN-γ. Furthermore, we did not investigate inhibitory mechanisms at a molecular level through techniques like western blotting to assess iNOS and cyclooxygenase-2 (COX-2) inhibition, or docking studies due to resource limitations. Despite these limitations, our study yielded positive outcomes, including the isolation of compounds 3, 5, 6, 9, 11, and 12 for the first time and the evaluation of their NO inhibitory activity from Vietnamese *M. thunbergii*. These novel findings will be the foundation for more comprehensive studies in the future.

## Ethics Statement

There were no ethical requirements for the collection and analysis of the data. All software used for the curation and analysis of the dataset were open source.

## CRediT authorship contribution statement

**Dao Cuong To:** Writing – review & editing. **Le Minh Hoang:** Conceptualization, Methodology, Data curation, Investigation, Visualization. **Hoa Thi Nguyen:** Conceptualization, Methodology, Data curation, Investigation, Visualization. **Truong Thi Viet Hoa:** Conceptualization, Methodology, Data curation, Investigation, Visualization. **Nhung Truong Thi Thuy:** Conceptualization, Methodology, Data curation, Investigation, Visualization. **Manh Hung Tran:** Writing – review & editing. **Phi Hung Nguyen:** Writing – review & editing. **Phuong Dai Nguyen Nguyen:** Writing – review & editing. **Ngu Truong Nhan:** Conceptualization, Methodology, Data curation, Investigation, Visualization. **Nguyen Thi Thu Tram:** Conceptualization, Methodology, Data curation, Investigation, Visualization.

## Data Availability

Quantitative NMR Data of Isolated Compounds (1-12) (Original data) (Mendeley Data)
